# Three dimensional modeling of atrioventricular valves provides predictive guides for optimal choice of prosthesis

**DOI:** 10.1038/s41598-022-10515-2

**Published:** 2022-05-06

**Authors:** Faizus Sazzad, Jin Hao Goh, Zhi Xian Ong, Zakaria Ali Moh Almsherqi, Satish R Lakshminarasappa, Kollengode R Ramanathan, Theo Kofidis

**Affiliations:** 1 Yong Loo Lin School of Medicine, National University of Singapore, Singapore, Singapore; 2 Department of Biological Sciences, National University of Singapore, Singapore, Singapore; 3 Department of Cardiac, Thoracic and Vascular Surgery, National University Heart Centre, Singapore, Singapore; 4 Centre for Translational Medicine, National University of Singapore, MD6, 14 Medical Drive, Level-8 (South), Singapore, 117599 Singapore

**Keywords:** Anatomy, Cardiology

## Abstract

Inaccuracies in intraoperative and preoperative measurements and estimations may lead to adverse outcomes such as patient-prosthesis mismatch. We aim to measure the relation between different dimensions of the atrioventricular valve complex in explanted porcine heart models. After a detailed physical morphology study, a cast of the explanted heart models was made using silicon-based materials. Digital models were obtained from three-dimensional scanning of the casts, showing the measured annulopapillary distance was 2.50 ± 0.18 cm, and 2.75 ± 0.36 cm for anterior and posterior papillary muscles of left ventricle, respectively. There was a significant linear association between the mitral annular circumference to anterior–posterior distance (p = 0.003, 95% CI 0.78–3.06), mitral annular circumference to interpapillary distance (p = 0.009, 95% CI 0.38–2.20), anterior–posterior distance to interpapillary distance (p = 0.02, 95% CI 0.10–0.78). Anterior–posterior distance appeared to be the most important predictor of mitral annular circumference compared to other measured distances. The mean length of the perpendicular distance of the tricuspid annulus, *a*, was 2.65 ± 0.54 cm; *b* was 1.77 ± 0.60 cm, and *c* was 3.06 ± 0.55 cm. Distance *c* was the most significant predictor for tricuspid annular circumference (p = 0.006, 95% CI 0.28–2.84). The anterior–posterior distance measured by three-dimensional scanning can safely be used to predict the annular circumference of the mitral valve. For the tricuspid valve, the strongest predictor for the circumference is the c-distance. Other measurements made from the positively correlated parameters may be extrapolated to their respective correlated parameters. They can aid surgeons in selecting the optimal prosthesis for the patients and improve procedural planning.

## Introduction

Atrioventricular heart valves, namely mitral and tricuspid valves, are prone to coexisting disease and may lead to heart failure. Mitral valve calcification and mitral valve prolapse are potentially life-threatening conditions. Concomitant tricuspid valve disease is not uncommon. Mitral and/or tricuspid intervention may entail increased procedural complexities^1,2^.

Treatment consists of atrioventricular (AV) heart valve replacement or valve repair for which a variety of interventional modalities exist^3^. Even mitral valve repair, the gold standard for resolving mitral regurgitation, encompasses a diversity of techniques. Furthermore, surgical procedures involved in the replacement of a diseased mitral/tricuspid valve are preceded by echocardiography and other imaging modalities^4,5^. However, inaccuracies arise during the surgical procedure when estimating the size of the AV valve orifice using surgical tools. Of note, circular, rigid valve prostheses are regularly used to replace a highly asymmetric and flexible annulus. Replacement of the valve would depend on the surgeon’s estimation based on the tool, which can lead to a deleterious mismatch.

The AV apparatus is highly heterogeneous. It consists of the annulus, the leaflets, chords, and the papillary muscles. Understanding of the AV apparatus’ morphology is critical, which determines surgical treatment, in order to avoid or mitigate ventricular failure and its systematic consequences^6^. Compared to the tricuspid valve, more problems are known to arise in the mitral valve. Consequently, more research is being done to improve and facilitate the treatment of the mitral valve. However, the tricuspid valve function is also an important determinant of survival^7^.

A silicon-based cast and a digital 3D representation of the model would allow easier manipulation to obtain a greater variety of measurements than simple two-dimensional measurement. 3D scanning technologies have been used as an effective method of constructing models as an anatomical representation of the human body^8,9^. This would also hope to provide a method for cross-referencing measurements obtained from echocardiography reconstruction of the mitral valve that may have inaccuracies from these imaging^10^.

To address the inaccuracies in AV surgery or therapeutic intervention arising from a variety of different treatment techniques and manual measurements during the surgical process, further analysis of the specific mitral valve dimensions that carry physiological significance should be explored. Understanding these dimensions could present certain patterns in the parameters that may help to improve the surgical process of AV valve interventions. While there exist multiple studies that have analyzed the AV valve and its related structures^11,12^, this study aimed to obtain measurements of the porcine atrioventricular valve in a three-dimensional (3D) space. We investigated the measurements of the anatomical features of the mitral and tricuspid of the explanted pigs’ heart using 3D scanning to better understand its architecture and provide a baseline for the relationship between the various measurements made in the mitral and tricuspid anatomy and the proposed anatomical reference.

## Materials and methods

This study was conducted at the Cardiac Surgery Research Laboratory at the National University of Singapore from January to November 2021. Data from all experiments were collected prospectively according to Office of Safety, Health and Environment (OSHE) guidelines with compliance to biological hazards tissue handling standard operating procedures at a Biosafety Level 3 Laboratory (BSL-3) facility. The study was approved by the Institutional Animal Care and Use Committee (IACUC), Centre for Life Sciences (CeLS), Department of Comparative Medicine (CM), Singapore (Protocol No #R17-1126), with all personnel handling hazardous materials, including research staff were informed of the risks and instructed on the handling methods. In total, thirty-six explanted swine heart samples were collected and enrolled for processing, while swine hearts with significant structural defects were excluded from our study.

### Sample size and distribution

Thirty-six explanted swine hearts were procured for processing, while hearts with significant structural defects, dissection, or traumatic injury were excluded from our study. There was four initial proof of concept experiments that were performed to establish the experiment setup. Sixteen explanted hearts were used for the morphological 2D analysis of AV valves. Six samples were used for Mitral valve complex assessment, and the rest ten were used for tricuspid valve complex evaluation, where the sample was used for one particular valve only due to extensive dissection of the samples. On the other hand, additional 16 samples were assigned to both AV valve casting after the initial four proof of concept studies. Out of Sixteen casting, all were useful for mitral valve evaluation, whereas two samples for tricuspid evaluation were discarded due to inadequate casting quality.

### Explanted swine heart models

**Figure 1:**
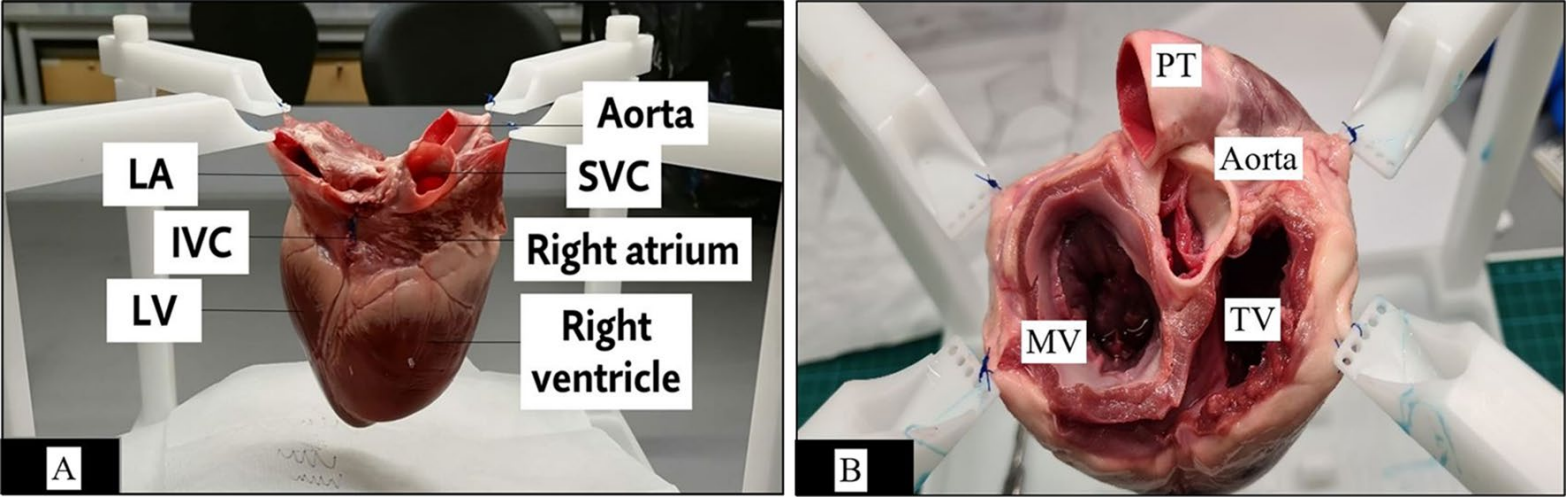
A customized, repositionable experimental holder is used to suspend the explanted swine heart by fixing it to the stand in the upper four sites with suture materials. (**A**) vertical orientation and side view of the explanted heart for casting. Atria was closed using 5/0 polypropylene suture materials permitting a small orifice to pour the liquid cast material. (**B**) Coronal orientation of the AV valves. *LA* left atrium, *IVC* Inferior vena cava, *SVC* Superior vena cava, *LV* Left ventricle, *MV* Mitral valve, *TV* Tricuspid valve, *PT* Pulmonary trunk.

In our study, we obtained casts of the cardiac chambers to yield better visualization of the cardiac atrioventricular apparatus. All great cardiac vessels—aorta, pulmonary trunk, superior vena cava (SVC), inferior vena cava (IVC), and pulmonary vein (PV) were partially removed from the heart. Blood clots and fibrin residues within the cardiac chambers were flushed out with water and/or removed surgically. Residual pericardium between the aortic root and pulmonary trunk (PT) was separated via blunt dissection. All valves (semilunar and atrioventricular valves) leaflets and their chords- were removed to ensure unobstructed flow of the cast material from atria to the ventricles. The explanted heart was suspended on a dedicated stand (Fig. 1A) with sutures used to anchor the four sites—LA, PV, RA, and the aorta in a free-floating position with the apex of the heart oriented inferiorly, allowing an uninterrupted flow of impression material and avoidance of artificial indentations forming on the cast. This enabled an accurate replica representation of the heart under diastolic conditions with avoidance of cavitation, pressure effects, and manipulation artifacts.

### 2D measurements of the AV valve

In the first step, we conducted 2D measurements of the AV valves. A different set of hearts (n = 16) was used for the 2D anatomical measurements. Following standard operating procedures in a wet lab setting, the samples were exposed by blunt dissection, using Metzenbaum scissors where the left and right atrium were removed (Fig. 1B). Before measurements were made, the last step of the dissection was to remove all leaflets and sub-valvular apparatus in both the mitral and tricuspid valves.

**Figure 2:**
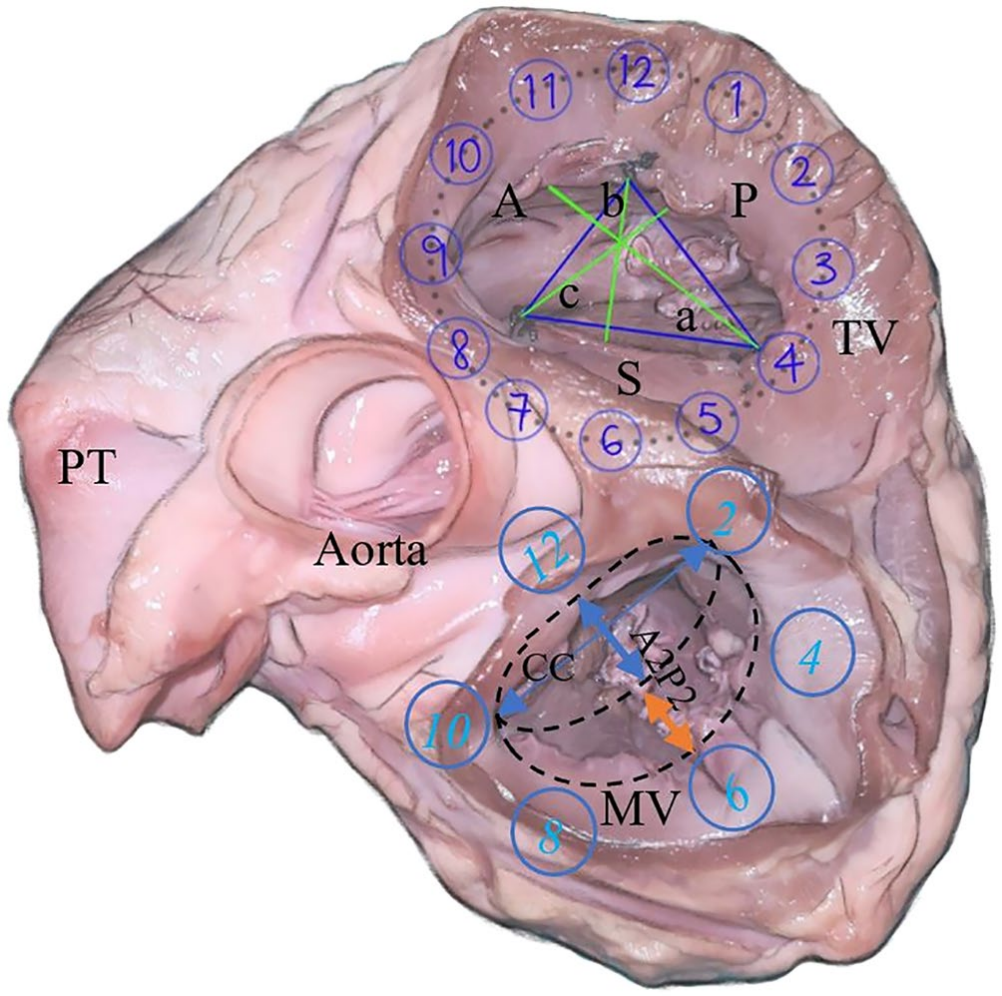
Anatomical orientation of the AV Valves clockwise. Mitral valve (MV) orientation (View from LA to LV): 10 o’clock and 2 o’clock are the respective commissures; 12 o’clock and 6 o’clock is the vertical distance between A2 and P2 segments. The tricuspid valve (TV) is marked at the annulus as S (Septal), A (Anterior), and P (Posterior), which represents the length of the corresponding annulus. The three commissures were positioned at 8 o’clock for Antero-septal, 4 o’clock for Postero-septal, and 12 o’clock for an Antero-posterior commissure. A perpendicular distance was measured from the commissure to the opposite annulus and perpendicular to the horizontal plane joining the other two commissure was marked as a, b, and c respectively, where “a” is a measured distance from 4 o’clock to 10 o’clock, “b” is a measured distance from 12 o’clock to 6 o’clock and “c” is a measured distance from 8 o’clock to 2 o’clock. *PT* Pulmonary trunk.

Due to the non-circular shape, planimetric variation, and small structure of the AV valve anatomy, a surgical suture (2/0 mersilk) was used to make various measurements to provide a more reliable and accurate curvature estimation. The annulus was first measured, followed by the respective commissures. These commissure points were marked out with a knot, with a surgical suture for references in measurement making. The shortest distance between the papillary muscle to each commissure was measured, as well as the perpendicular and horizontal distances of the tricuspid, as shown by the image in Fig. 2. These points were arranged based on the anatomical clock positions, with respect to a surgical orientation of the mitral and tricuspid valve during surgery. In the second round of dissection, the coronal section of the dissected heart is observed. The measurements for the height of the papillary muscle, distance from annulus ring to papillary tip, and ventricle depth were obtained. The measurement method is added to the Supplementary Doc 1.

### The casting of impression materials

**Figure 3:**
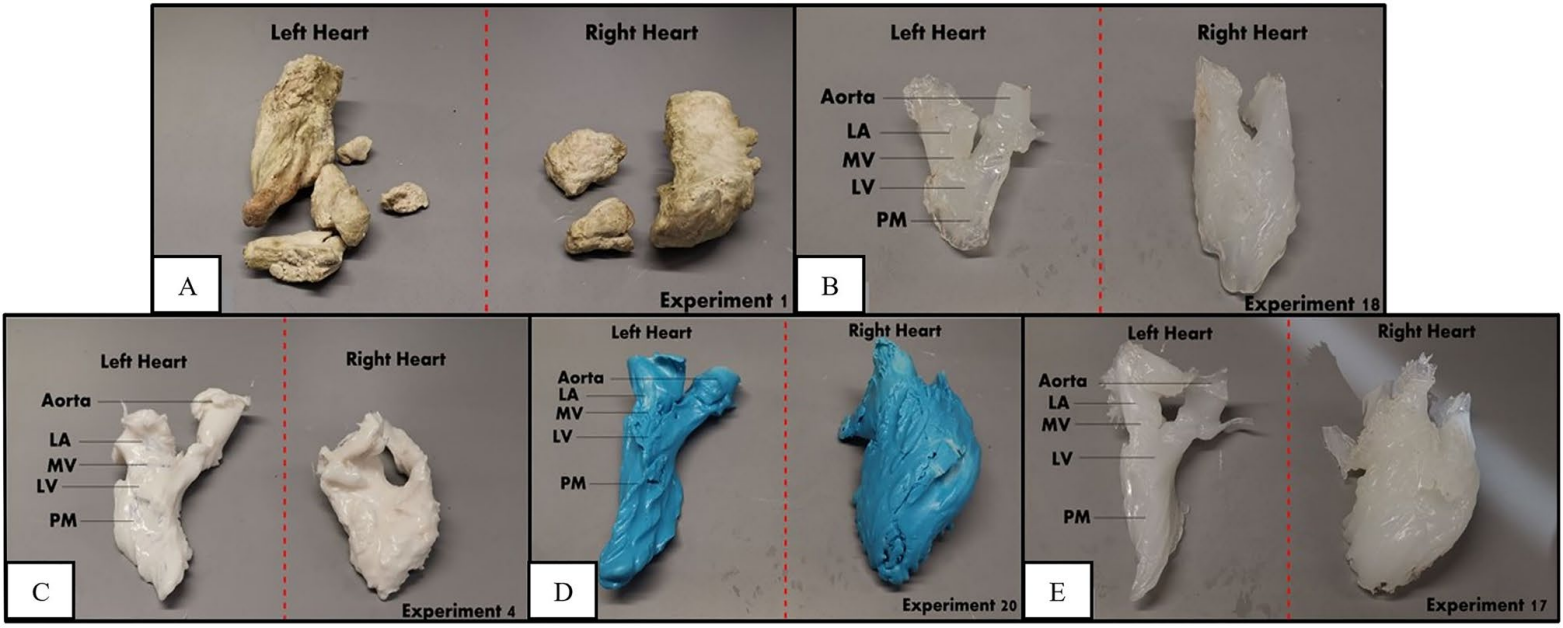
Figure panel showing the castings of AV chambers (both right and left) with the use of different impression materials: (**A**) Gypsum cement (LUNABEAN), (**B**) EVA (Ethylene–vinyl acetate) copolymers (STANLEY), (**C**) RTV (Room-temperature-vulcanizing) silicone, (**D**) EASYMOULD silicone rubber, (**E**) DRAGONSKIN silicone rubber.

We utilized five different cast materials—Gypsum cement (LUNABEAN) (Fig. 3A), EVA (Ethylene–vinyl acetate) copolymers (STANLEY) (Fig. 3B), RTV (Room-temperature-vulcanizing) silicone (Fig. 3C), Silicone rubber from EASYMOULD (Fig. 3D), and DRAGONSKIN (Fig. 3E) for a total of 20 explanted swine heart experiments. Each material was prepared according to the recommended instructions prior to installation; then were instilled into the heart via the LA and RA openings with a 30 ml introducer syringe or via direct casting in accordance with its unique material viscosity (Supplementary Doc 1). The luer lock tip of the introducer syringe was modified to enlarge the opening for smooth delivery of impression material into the heart. The heart filled with impression materials was kept in position for 45–60 min for sedimentation then placed into a fridge for 24 h at 5.9 °C subjective for consolidation. The cast was removed from the heart via incisions along the left and right cardiac borders on the next day to obtain a cast (Fig. 3A–E). The casts are excised from the heart, and excess materials were trimmed. Anatomical structures like MV and PM were located and marked on the cast.

### Creation of 3D models

The casting models were scanned using the EinScan Pro 2 × Plus 3D Scanner from the Shining 3D Pte Ltd under the ownership of the Technology Enhanced Learning (TEL) Imaginarium. The scanner uses structured-light 3D technology to obtain the geometry of the scanned subject. The scanner projects light onto the object, which is reflected into the camera. The light is distorted along the surface of the 3D object, which is captured by cameras located at a perspective different from that of the projected light, allowing for the geometric reconstruction of the object (Supplementary Fig. 1).

The casting models were placed on a turntable, and the exposure was adjusted through the EXScan Pro software from Shining 3D under an LED light source to ensure adequate lighting. Exposure was a fixed variable, and the number of steps for the turntable was set to eight before allowing the scan to proceed. Each step is a rotation of the turntable, indicating that after every step, the model will be rotated at an angle of 45° for the scanner to scan a new face of the model to provide an accurate representation of the model. Two-dimensional measurements were obtained in centimeters, while the outline of the mitral valve annulus was displayed as a 3D figure. With the turntable, the scanner has a range error for its scan accuracy of up to 0.04 mm. The scanner also has a slice resolution of 0.16 mm, which was sufficient as the visible details in the models, such as the papillary muscle tips, have a distance greater than 0.16 mm.

Our standard protocol started with LV first. The LV cast was placed on the turntable with the anterolateral papillary muscle facing up to allow the scanner to record the top layer of the model. The model was then flipped 180° and scanned again to record the bottom layer. The two layers were then aligned through EXScan Pro. The apex of the heart, the tip of the left atrium (LA), and the tip of the aorta on the model were used as points to align the two layers together. The final product was then exported and saved as a Wavefront file. The same procedure was followed for RV. The right heart models were placed with their medial, septal, sides facing up first before rotating the model 180° to have its lateral side facing up. For the remaining, the same steps were repeated in order to obtain the scans. Alignment of top and bottom scans presents a measurement risk, as it was done by eyeball estimation. In order to reduce the inaccuracy, three different scans of each model were obtained, and the average measurements of the three scans were calculated for further analysis.

### Computational measurements

In our study, for computational measurement, we have used BLENDER, an open-source 3D creation software, which also has the capabilities of modeling 3D objects, and can import 3D scans of various formats along with obtaining measurements for the objects. Using Blender, different sets of data were obtained from the Wavefront model scan file. The distance between the tip of the LA and the heart apex of the cast model was measured to ensure that the digital model is scaled to the correct size in Blender. After importing the Wavefront file into Blender, the model was scaled to match the appropriate size. The measure tool in Blender was then used to measure the distances from the points plotted on the model. Each 3D model was measured three times to eliminate inaccuracies and averaged.

**Figure 4:**
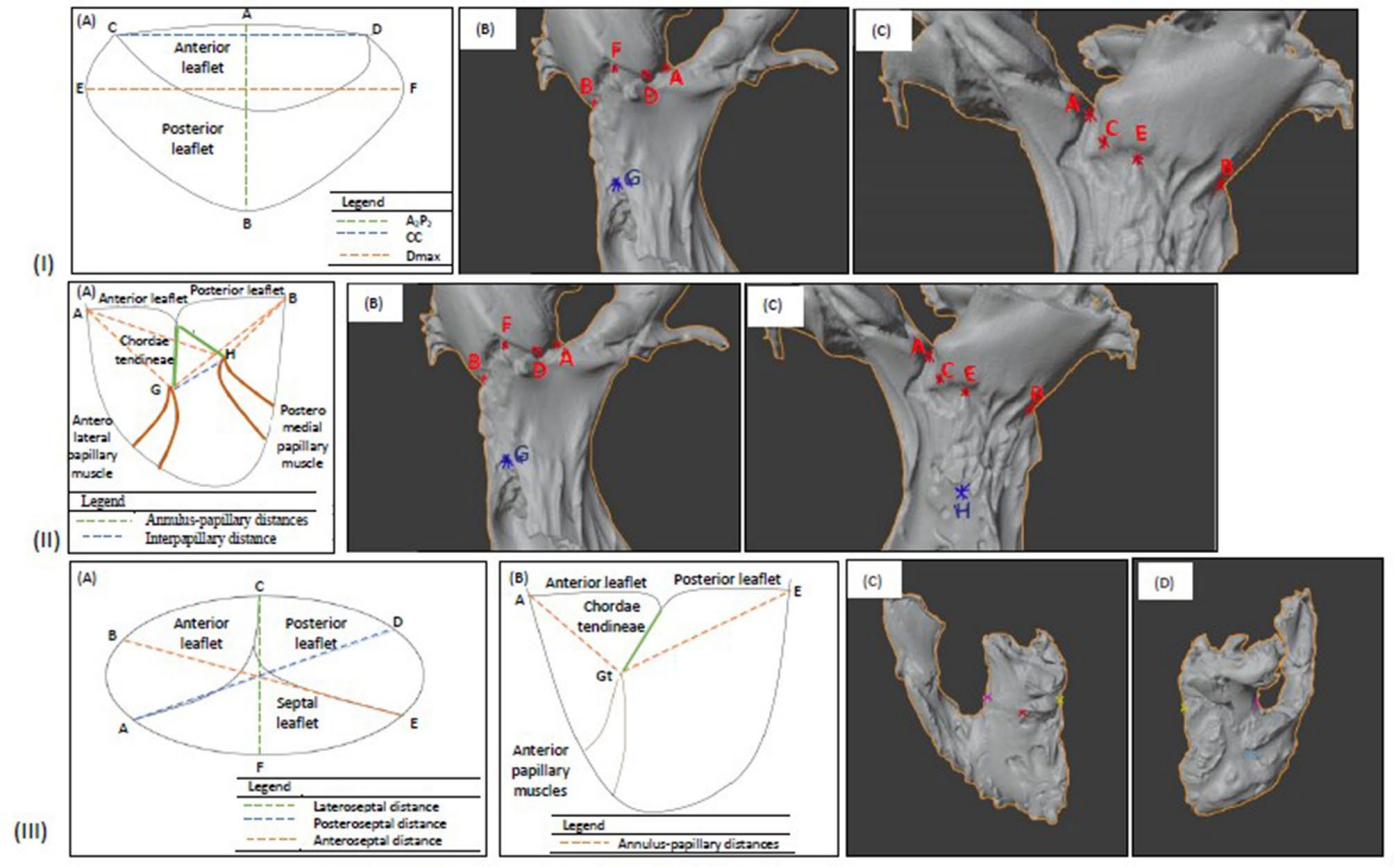
Schematic diagram and computational images extracted from blender showing dimensions of interest. (**I**) (**A**). Diagram representation of the mitral valve viewed from the LA (Axial View). The distance from A to B refers to the anteroposterior diameter (A_2_P_2_), the longest diameter when measured in an anteroposterior direction. The distance from C to D refers to the inter-commissural distance (CC), or the diameter between the locations where the anterior and posterior leaflets meet. The distance from E to F refers to the diameter maximum (D_max_), which is the longest diameter when measured in a lateromedial fashion; (B) Anterior view of model showing points A, D, F, and B along with the mitral valve, with reference to (**I**(A)); (C) Posterior view of model showing points A, C, E, and B along with the mitral valve, with reference to (**I**(A)). (**II**) Papillary muscles in relation to the mitral valve in sagittal view. (A) Cross-sectional representation of left ventricle including mitral valve complex. G refers to the tip of the anterolateral papillary muscle, while H refers to the posteromedial papillary muscle. The distance from G to H is the interpapillary distance (I_PAP_), or the distance between the tips of the papillary muscles, was measured. The distances between the points A, B, C, D, E, and F [as in Figure (**I**(A))] to the tips of both papillary muscles. These measurements were the various annulus-papillary distances. (B) Anterior view of the model shows the tip of the anterolateral papillary muscle. The single highest point of the indentation from the papillary muscle was taken as point G. (C) Posterior view of the model showing the tip of the posteromedial papillary muscle. The single highest point of the indentation from the papillary muscle was taken as point H. **III**(A) Diagram representation of the tricuspid valve axial view (viewed from the RA). The three commissures were positioned at A for Antero-septal, E for Postero-septal, and C for Antero-posterior commissure. A distance was measured from the commissure to the opposite annulus was marked as AD, EB, and CF, representing a,b,c, respectively (as in Fig. 2). (B) Antero-lateral papillary muscle in relation to the tricuspid valve in sagittal view. Cross-sectional representation of right ventricle including tricuspid valve complex. Gt refers to the tip of the anterolateral papillary muscle. The distances between points A and E, [as in Figure (**III**(A))], were measured to the tip of the papillary muscle.

The draw tool was used to create an outline of both mitral and tricuspid annulus, and the circumference was obtained under the Curve Info. Depiction of the points of interest used to obtain the measurements is shown in Fig. 4. The distances from the points indicated in Fig. 4I(A–C) and the papillary muscles were measured, along with the interpapillary distance for the mitral valve in Fig. 4II(A).

MV measurements included: Annulus circumference (AC), Annulus 3D structure, Inter-commissural diameter (CC), Anteroposterior diameter (A_2_P_2_), Diameter maximum (D_max_), Interpapillary distance (I_PAP_). We have also measured the annulus to corresponding papillary muscle tip distance (APD), as shown in Fig. 4I(A) and II(A). Annulus to papillary distance (ALP) at C(10)G marking represents anterolateral commissure at 10 o’clock (Fig. 2) to the tip of corresponding papillary muscle H. Similarly, the annulus to papillary distance (PMP) for posteromedial commissure at 2 o’clock (Fig. 2) to the tip of the papillary muscle distance was taken from D(2)H point. Additionally, Papillary muscles measurements the I_PAP_ (G to H) and the distances of the ALP (annulus-papillary distances) to points A, B, C, D, E, and F along the mitral valve [Fig. 4I(A–C)] shows the distributions of the distances measured. With reference to ALP, the annulus-papillary distances measured from A(12)G, B(6)G, C(10)G, D(2)G, E(9)G, and F(3)G. With reference to PMP, the different means for annulus-papillary distances to points along the mitral valve include from A(12)H, B(6)H, C(10)H, D(2)H, E(9)H, and F(3)H [Fig. 4II(A–C)].

For the Tricuspid valve, we assessed: Annulus circumference (AC), Septal annulus length (SA), Anterior annulus length (AA), Posterior annulus length (PA). The imaginary triangle joining the three commissures (Figure-2) was used for horizontal distance measurement. The distance from 12 o’clock (Antero-posterior commissure) to 8 o’clock (Antero-septal commissure) was represented as HA(12–8); distance from 8 o’clock (Antero-septal commissure) to 4 o’clock (Postero-septal commissure) to was represented as HS(8–4), and distance from 4 o’clock (Postero-septal commissure) to 12 o’clock (Antero-posterior commissure) to was represented as HP(4–12). A perpendicular distance was measured from the corresponding commissure to the opposite annulus, which was perpendicular to the horizontal plane joining the other two commissure was marked as a, b and c respectively. “a” was a measured distance from 4 o’clock to 10 o’clock, “b” is a measured distance from 12 o’clock to 6 o’clock and “c” is a measured distance from 8 o’clock to 2 o’clock (Fig. 2). Annulus-papillary distances (APD) were also measured from A(8)Gt, B(10)Gt, C(12)Gt, D(2)Gt, E(4)Gt, and F(6)Gt as shown in Fig. 4III(A–D).

### Statistical analysis

The Statistical Package for the Social Sciences (version 27.0) was used for interactive and batched statistical analysis. Categorical data were presented in the frequency tables and one-way analysis of variance (ANOVA) analysis was performed where applicable. Multiple linear regression analysis was performed to identify the correlation among the interest variables. Statistical significance was defined as p-value < 0.05.

### Institutional review board

This study was approved by the Institutional Animal Care and Use Committee (IACUC), Centre for Life Sciences (CeLS), Department of Comparative Medicine (CM), National University of Singapore (Reference #R17-1126).

### Informed consent

Preclinical, experimental study, informed consent is not applicable.

## Results

### Morphology of AV valve complex: 2D measurements

#### Mitral valve

The mean mitral annular circumference (AC) was 10.07 ± 0.49 cm [9.20–10.50 cm]; mean inter-commissural distance (CC) was 3.12 ± 0.21 cm [2.70–3.30 cm]; mean antero-postero diameter (A_2_P_2_) was 2.62 ± 0.26 cm [2.30–2.90 cm]; mean maximum annular diameter (D_max_) was 3.52 ± 0.22 cm [3.30–3.80 cm]. The distance between the two papillary muscle tip (I_PAP_) was 1.5 ± 0.21 cm [1.30–1.80 cm].

ALP distance for C(10)G represents anterolateral commissure at 10 o’clock (Fig. 2) to the tip of papillary muscle H. The mean C(10)G distance was 2.50 ± 0.18 cm [2.20–2.70 cm] in our series. Similarly, the PMP distance for posteromedial commissure D(2)H was 2.75 ± 0.36 cm [2.20–3.10 cm]. The measured LV free wall thickness was 1.43 ± 0.21 cm [1.20–1.80 cm] with a LV radius 2.78 ± 0.18 cm [2.60–3.10 cm] and LV Height measured was 2.82 ± 0.32 cm [2.50–3.30 cm].

#### Tricuspid valve

The mean tricuspid annular circumference (AC) was 8.96 ± 1.49 cm [7.27–11.80 cm]; mean septal annulus length (SA) was 2.73 ± 0.89 cm [1.63–4.47 cm]; mean anterior annular length was 3.90 ± 0.38 cm [3.32–4.50 cm], and mean posterior annulus length was 2.33 ± 0.58 cm [1.43–3.02 cm]. From the data obtained, the posterior annulus (PA) was the shortest section of the annulus ring, as seen in the average, minimum and maximum measurements. The tricuspid valve complex of the swine model showed that the anterolateral papillary muscle is the most prominent and identifiable, while the other two papillary muscles were rudimentary. In some cases, the anterior leaflet and part of the septal leaflet chords were found directly attached to the ventricular septum.

The mean APD distance at A(8)Gt was 2.87 ± 1.38 cm [2.07–5.57 cm] in our series. Similarly, APD distance at C(12)Gt was 1.94 ± 0.77 cm [1.37–4.00 cm], and APD distance at E(4)Gt was 1.99 ± 1.61 cm [0.98–5.30 cm]. From the data obtained, the A(8)Gt was the longest distance from its papillary muscle to its respective commissure as seen in the average, minimum and maximum measurements. There were no definite trends between the C(12)Gt and E(4)Gt. The mean horizontal distance from commissure to opposite commissure HA(12–8) was 2.55 ± 0.58 cm [1.80–3.39 cm]. Similarly mean distance of HS(8–4) was 2.94 ± 0.43 cm [2.43–3.67 cm], and mean distance of HP(4–12) was 2.25 ± 0.24 cm [1.95–2.69 cm]. From the data obtained, the longest horizontal distance of the corresponding position was from the HS as seen in the average, minimum and maximum measurements. There were no definite trends between HA and HP. The mean length of perpendicular distance a was 2.65 ± 0.54 cm [1.98–3.67 cm]; mean length of b was 1.77 ± 0.60 cm [1.06–2.91 cm], and mean length of c was 3.06 ± 0.55 cm [2.36–3.86 cm]. From the data obtained, the longest perpendicular distance from the corresponding position was from the (4–10) as seen in the average, minimum and maximum measurements. The shortest perpendicular distance from the corresponding position would be from the c(8–2).

Three measurements of the papillary muscle were taken for height, width, and thickness. The height ranges from 2.0 to 3.7 cm. The width ranges from 1.5 to 2.2 cm. The thickness ranges from 0.3 to 0.7 cm. The height of the papillary was the most diverse dimension. The thickness of the papillary muscle was the most consistent among the samples measured.

### 3D modeling and cast measurements

#### Mitral valve

**Figure 5:**
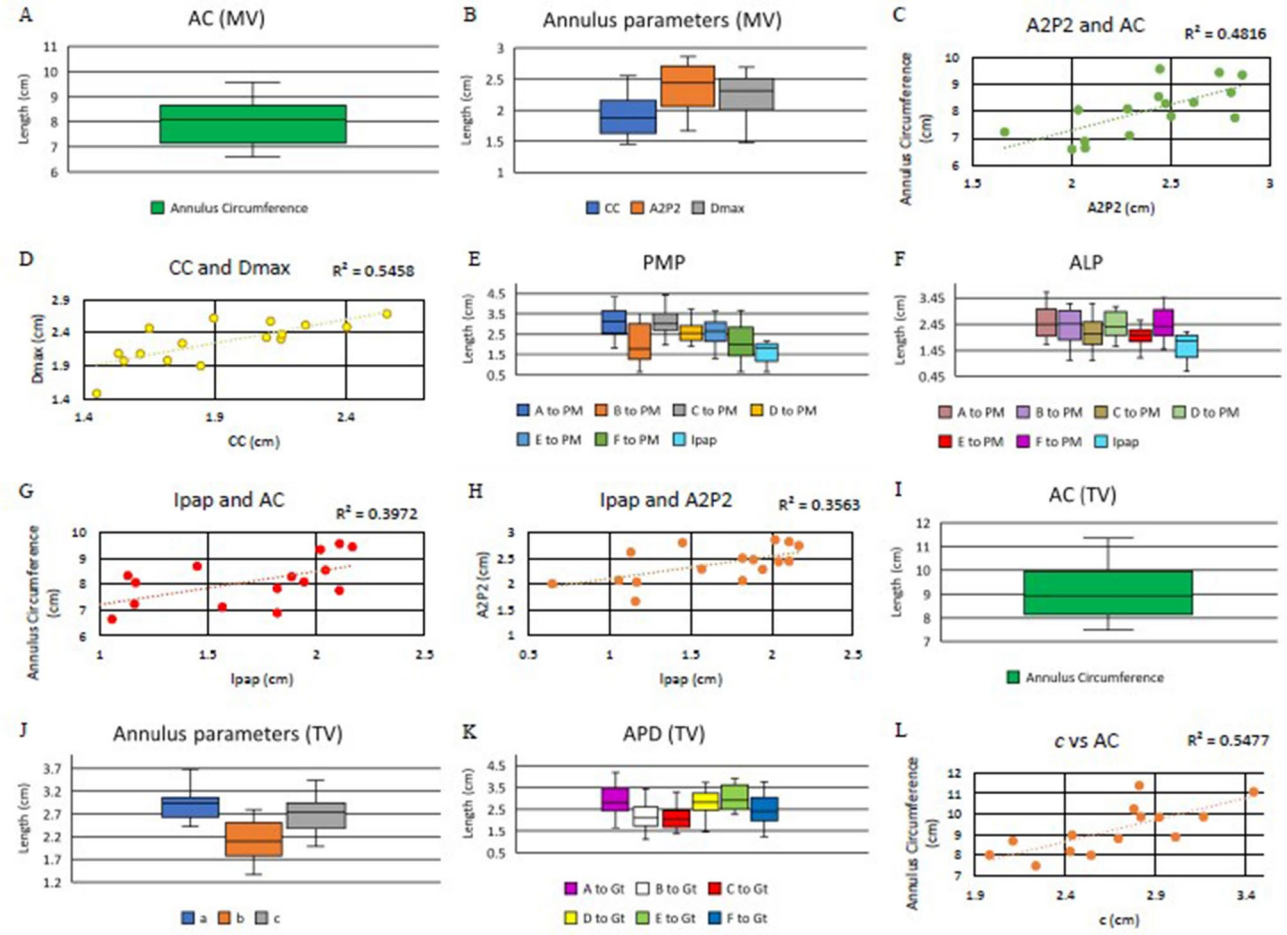
Mitral valve (**A**–**H**), Tricuspid valve (**I**–**L**). (**A**) Box and whisker plots of mitral valve annulus parameters in Annulus circumference (AC); (**B**) Inter commissural distance (CC); (**C**) Vertical distance of mitral valve (A_2_P_2_); the maximum transverse diameter of the mitral valve (D_max_); (**D**) Correlation between CC and D_max_ diameters of the mitral valve; (**E**) Distribution of annulus-papillary distances for posteromedial papillary muscle (PMP); (**F**) Distribution of annulus-papillary distances for anterolateral papillary muscle (ALP) and Interpapillary distance (I_PAP_); (**G**) Correlation between I_PAP_ and AC; (**H**) Correlation between I_PAP_ and A_2_P_2_; (**I**) Box and whisker plots of tricuspid valve annulus parameters in Annulus circumference (AC); (**J**) Commissural lengths, Septal annulus (SA), Anterior annulus (AA), posterior annulus (PA); (**K**) Distribution of annulus-papillary distances; (**L**) Correlation between perpendicular distance c (8–2) and AC.

The distributions of the various parameters are displayed in Fig. 5A,B. Figure 5C,D showed the correlations between the various mitral valve complex parameters also show that there was a positive correlation between A_2_P_2_ vs. mitral annulus circumference (AC) (R^2^ = 0.48) and CC vs. D_max_ (R^2^ = 0.55). Box and whisker plots of I_PAP_ and annulus-papillary distances showed a mean I_PAP_ of 1.82 ± 0.21 cm.

With reference to ALP, the annulus-papillary distances have varying means when measured from A(12)G (3.08 ± 0.67 cm), B(6)G (1.97 ± 0.97 cm), C(10)G (3.07 ± 0.64 cm), D(2)G (2.52 ± 0.61 cm), E(9)G (2.62 ± 0.69 cm), and F(3)G (2.08 ± 0.83 cm) (Fig. 5E). With reference to PMP, the different means for annulus-papillary distances to points along the mitral valve include from A(12)H (2.51 ± 0.59 cm), B(6)H (2.33 ± 0.70 cm), C(10)H (2.14 ± 0.61 cm), D(2)H (2.40 ± 0.61 cm), E(9)H (1.96 ± 0.48 cm), and F(3)H (2.41 ± 0.61 cm) (Fig. 5F).

The Simple and multiple linear regressions of dimensions in the mitral valve were analyzed and displayed in Table 1. There was a significant linear association between mitral AC-A_2_P_2_ (p = 0.003), AC-I_PAP_ (p = 0.009) (Fig. 5G), A_2_P_2_-I_PAP_ (p = 0.02) (Fig. 5H), and D_max_-CC (p = 0.001). There was a 1.65 cm (95% CI 0.36–2.94) increase in the mean length of mitral AC for each cm A_2_P_2_ (p = 0.006). A_2_P_2_ appears to be the ‘most important’ predictor for Mitral AC, compared to CC, D_max,_ and I_PAP_. The rest of the correlations were statistically non-significant, and correlation analysis has been added to Supplementary Doc 1 (Supplementary Figs. 2–4).

**Table 1:** Simple and multiple linear regression of parameters for both the mitral and tricuspid valve.

	DV	IV	R^2^	ANOVA-F	Co-B	p	L-95	U-95
**SLR**								
MV	AC	CC	0.0008	0.91	0.08	0.91	− 1.57	1.75
	AC	A_2_P_2_	0.48	0.003*	1.92	0.003*	0.78	3.06
	AC	D_max_	0.0002	0.96	0.04	0.96	− 1.7	1.74
	AC	I_PAP_	0.40	0.009*	1.29	0.009*	0.38	2.20
	A_2_P_2_	CC	0.04	0.47	− 0.20	0.47	− 0.79	0.38
	A_2_P_2_	D_max_	0.15	0.14	− 0.42	0.14	− 0.99	0.15
	A_2_P_2_	I_PAP_	0.36	0.02*	0.44	0.02*	0.10	0.78
	D_max_	CC	0.54	0.001*	0.72	0.001*	0.34	1.09
	D_max_	I_PAP_	0.17	0.11	− 0.29	0.11	− 0.64	0.07
	CC	I_PAP_	0.11	0.21	− 0.24	0.21	− 0.62	0.15
TV	AC	*a*	0.35	0.03*	1.91	0.03*	0.26	3.57
	AC	*b*	0.26	0.06	1.33	0.06	− 0.08	2.74
	AC	*c*	0.55	0.002*	2.12	0.002*	0.91	3.33
	*a*	*b*	0.10	0.26	0.26	0.26	− 0.22	0.73
	*c*	*b*	0.16	0.15	0.37	0.15	− 0.15	0.89
	*c*	*a*	0.14	0.19	0.32	0.19	− 0.19	0.84
**MLR**								
MV	AC	CC	0.59	0.006*	− 0.15	0.84	− 1.74	1.43
		A_2_P_2_			1.65	0.01*	0.36	2.94
		D_max_			1.41	0.09	− 0.31	3.13
		I_PAP_			0.93	0.06	− 0.03	1.89
	I_PAP_	A (PMP)	0.20	0.09	0.44	0.04	0.01	0.87
		A (ALP)			− 0.14	0.55	− 0.63	0.35
	I_PAP_	B (PMP)	0.25	0.06	− 0.06	0.67	− 0.34	0.22
		B (ALP)			0.44	0.03	0.07	0.83
	I_PAP_	C (PMP)	0.27	0.07	0.49	0.03	0.07	0.92
		C (ALP)			− 0.22	0.31	− 0.66	0.23
	I_PAP_	D (PMP)	0.21	0.08	0.62	0.03	0.06	1.18
		D (ALP)			− 0.43	0.21	− 1.13	0.27
	I_PAP_	E (PMP)	− 0.08	0.67	0.15	0.44	− 0.26	0.56
		E (ALP)			− 0.17	0.54	− 0.77	0.42
	I_PAP_	F (PMP)	0.0004	0.39	0.07	0.79	− 0.52	0.67
		F (ALP)			0.19	0.61	− 0.61	0.99
TV	AC	*a*	0.60	0.006*	1.06	0.12	− 0.36	2.47
		*b*			0.48	0.37	− 0.66	1.62
		*c*			1.56	0.02*	0.28	2.84

*Significant; *SLR* Simple linear regression, *MLR* Multiple linear regression, *DV* Dependent variable, *IV* Independent variable, *AS-F* ANOVA significance-F, *Co-B* Coefficient B, *L-95* Lower 95%, *U-95* Upper 95%, *TV* Tricuspid valve, *MV* Mitral valve, *AC* Annular circumference, *CC* Inter-commissural distance, *I*_*PAP*_ Inter-papillary distance, *D*_*max*_ Maximum diameter of the mitral valve, *A*_*2*_*P*_*2*_ Anteroposterior diameter of the mitral valve.

#### Tricuspid valve

The distributions of the various parameters are displayed in Fig. 5I,J. Box and whisker plots of annulus-papillary distances (APD) showed the dimensions have varying means when measured from A(8)Gt (2.91 ± 0.72 cm), B(10)Gt (2.28 ± 0.81 cm), C(12)Gt (2.19 ± 0.69 cm), D(2)Gt (2.82 ± 0.63 cm), E(4)Gt (3.04 ± 0.54 cm), and F(6)Gt (2.48 ± 0.76 cm) (Fig. 5K).

Figure 5L showed that there was a positive correlation between tricuspid annulus circumference (AC) to perpendicular distance “*c*” (R^2^ = 0.54). The tricuspid AC had a linear association with perpendicular distances “*a*” and “*c*” (Table-1). There was a 1.56 cm (95% CI 0.28–2.84) increase in the mean length of tricuspid AC for each cm “*c*” (p = 0.02). The perpendicular distance “*c*” appears to be the ‘most important’ predictor for tricuspid AC. The APD measured from five definitive points of the tricuspid annulus has shown no significant correlation. The rest other correlations for the tricuspid valve were not significant statistically, and the correlation analysis is added in the Supplementary Doc 1 (Supplementary Figs. 5, 6).

## Discussion

This study showed significant positive correlations in certain mitral and tricuspid valve dimensions. Our main findings were the significant association between mitral AC-A_2_P_2_, AC-I_PAP_, A_2_P_2_-I_PAP_, and D_max_-CC, out of which A_2_P_2_ was the most important predictor of mitral AC. Additionally, the perpendicular distance-*c* was the most significant predictor for tricuspid AC. As such, this would allow surgeons to better extrapolate from a single measurement of the mitral and/or tricuspid valve to determine the optimal prosthesis size.

In the mitral valve, a larger A_2_P_2_ denotes a possibly larger annulus circumference. The tricuspid valve had a relatively less distinct annulus compared to the mitral valve. The intraoperative finding of a larger distance-*c* may reflect the need for a larger valve size or the need to reduce the annular size via annuloplasty. These correlations show both the normal anatomical relationship and the ideal size for treatment—having an equation may aid the interventional cardiologist or surgeon in planning procedures. However, the heart is a physically dynamic organ—these anatomical relationships need to cautiously correlate with hemodynamic performance. In our setting, the heart was examined under diastolic conditions.

The morphology of the swine heart bears a close resemblance to the human heart. The mitral and tricuspid valves are similar to the human heart with respect to the size, leaflets, and chordae tendineae layout^13^. Porcine heart models are therefore suitable as an alternative in heart valve experimentations. Dimensions of the atrioventricular valve leaflets were not obtained in this study as they were presumed to be diseased and removed. Additionally, we have noticed about ~ 10–15% reduction in actual physical size and distance of a measured dimension in a real specimen versus a cast.

Feasibility studies of 3D valvular modeling in interventional and surgical planning have been previously performed^14,15^. Patients required multi-modal and multiple computed tomographies and 3D echocardiography for valvular delineation prior to printing of the models. Issues encountered include poor cost-effectiveness, and limited clinical utility in routine valvular interventions. Furthermore, the resolution of images obtained may skew the evaluation of the valves. 3D echocardiography is operator-dependent and may encounter loss of signal, blurring, or partial acquisition of the valve^14^.

Accurate anatomical modeling was achieved through 3D cast models. True quantitative measurements were reproduced via hand-held 3D scanners. The delineation of the valvular structures and quantitative measurements guide the understanding of relationships between valvular dimensions. Hence, the anatomical relationships elicited may have a clinical role in planning for interventions in each unique individual’s anatomy. The 3D model in this study is in accordance with the shape of the MV annulus cited in other studies^15–18^. The MV annulus depicted in the model is represented by a non-planar saddle-shaped figure (Supplementary Fig. 7). Additionally, other studies provide a similar morphology of the model of the TV as in this study^18–21^.

Moreover, the construction of a 3D model would have possible applications as a tool for anatomical education. Due to the non-planar structure of both the MV and TV, their morphology can be better understood through a 3D model that would be able to better capture the shape along the x, y, and z axes. 3D models show promising results as a form of education in guiding students compared to cadaveric models^21–24^. Furthermore, virtual 3D models would present greater efficiency and accessibility to anatomical research. Virtual programs are capable of displaying morphological aspects through animations^25^, providing greater interactivity. Understanding the MV and TV morphology through efficient education and research can produce clinical applications with more accurate biomimicry and aid the choice of the prosthetic valve. One of the major limitations of this study is the small pool of samples. The positioning of the heart to stimulate the diastolic state may not be ideal in ensuring accurate measurements due to the flaccidity of the heart. However, the standardized positioning and anchors on the heart helped to account for the variation in positioning.

In addition, With the abnormal TV and MV, the normal morphology and shape of both annuli are always disturbed. Due to this distortion of the shape, the diameters also are changed dramatically. However, AV valve pathology comes with multi-fold changes: normal annuli, dilated annuli, asymmetrically dilated annuli, calcification annuli, and more. Regretfully, no size evaluation can capture the full cycle of the heart, the individual patients’ annular variability, and pathology, thus altering the level of mismatch. A better, rather functional metric, which may correlate better with the average EOA of the valve, following implantation. The principle of implanting the biggest possible valve is best served by a proper anatomical measurement rather than a rigid circular, pre-sized tool. In short, our proposed assessment method is not perfect but the least flawed amongst them all. This is a limitation in the current study; further 3D modeling studies on experimental diseased hearts or in vitro heart models would be needed to validate these findings.

## Conclusion

The 3D analysis of the morphological variables of the atrioventricular heart valve complex will provide a deeper understanding of mitral and tricuspid valve morphology in the surgical context and can guide decision-making. This will allow surgeons and interventional cardiologists to extrapolate from a single measurement of the mitral and/or tricuspid valve to the necessary dimensions of the mitral and or tricuspid valve implants.

## Supplementary Information

Supplementary Information.

## Data Availability

The authors confirm that the data supporting the findings of this study are available within the article and its supplementary materials. Raw data were generated at Cardiac Surgery Research Laboratory at the National University of Singapore. Derived data supporting the findings of this study are available from the corresponding author on request.

## Abbreviations

3D: Three dimensional
AC: Annulus circumference
AAC: Total annular circumference
ANOVA: Analysis of variance
AV: Atrioventricular
BSL-3: Biosafety level 3 laboratory
CeLS: Centre for life sciences
CM: Comparative medicine
EVA: Ethylene–vinyl acetate
EOA: Effective orifice area
IACUC: Institutional animal care and use committee
IVC: Inferior vena cava
LA: Left atrium
LED: Light emitting diode
LV: Left ventricle
MV: Mitral valve
OSHE: Office of safety, health and environment
PT: Pulmonary trunk
PM: Papillary muscle
PV: Pulmonary vein
RA: Right atrium
RTV: Room-temperature-vulcanizing
RV: Right ventricle
SMC: Superior vena cava
SOP: Standard operating procedure
TEL: Technology enhanced learning
TMVR: Transcatheter mitral valve replacement
TV: Tricuspid valve
