# Supplementary material for: Three dimensional modeling of atrioventricular valves provides predictive guides for optimal choice of prosthesis
Source: Sci Rep. 2022 May 6;12:7432. doi: 10.1038/s41598-022-10515-2 (PMC9076597; doi:10.1038/s41598-022-10515-2)
Supplement: Supplementary file 1 — Supplementary Information. [file 41598_2022_10515_MOESM1_ESM.docx]

**Definitions of studied Dimensions and metrics**

**AC:** Annular Circumference of the atrioventricular heart valves.

**CC:** Inter-commissural distance of the mitral valve (*10 o’clock and 2 o’clock are the respective commissures; C to D in figure-5I refers to the CC or the diameter between the locations where the anterior and posterior leaflets meet)*

**A_2_P_2_:** Antero-posterior diameter of the mitral valve (*12 o’clock and 6 o’clock is the vertical distance between A2 and P2 segments; A to B in figure-5I refers to the* A_2_P_2_ *diameter)*

**D_max_:** Maximum annular diameter of the mitral valve (*E to F in figure-5I refers to the D_max_, which is the longest diameter when measured in a lateromedial fashion)*

**I_PAP_:** Interpapillary distance of the left ventricle (*The distance from G to H, is the I_PAP_, in figure-5II)*

**AS:** Antero-septal commissure of the tricuspid valve *(8 o’clock position)*

**PS:** Postero-septal commissure of the tricuspid valve *(4 o’clock position)*

**AP:** Antero-posterior commissure of the tricuspid valve *(12 o’clock position)*

**SA:** Septal annulus of the tricuspid valve *(measured length of the septal annulus)*

**AA:** Anterior annulus of the tricuspid valve *(measured length of the anterior annulus)*

**PA:** Posterior annulus of the tricuspid valve *(measured length of the posterior annulus)*

**Perpendicular distance:** Perpendicular distance at the level of the tricuspid valve annulus is measured from the commissure to the opposite annulus and the line is perpendicular to the horizontal plane joining the other two commissures (Figure-2).

- *“a”(4-10) is a measured distance from 4 o’clock to 10 o’clock*
- *“b”(12-6) is a measured distance from 12 o’clock to 6 o’clock*
- *“c”(8-2) is a measured distance from 8 o’clock to 2 o’clock*

**Horizontal distance:** Horizontal distance at the level of tricuspid valve annulus measured from one commissure to the opposite commissure and the line was drawn to grate an imaginary triangle (Figure-2).

- HA (12-8): Vertical distance from 12 o’clock (Antero-posterior commissure) to 8 o’clock (Antero-septal commissure) was represented as VA (12-8)
- HS (8-4): Vertical distance from 8 o’clock (Antero-septal commissure) to 4 o’clock (Postero-septal commissure) to was represented as VS (8-4)
- HP (4-12): Vertical distance from 4 o’clock (Postero-septal commissure) to 12 o’clock (Antero-posterior commissure) to was represented as VP (4-12)

**APD:** Annulus-papillary distance (measured distance from a point at the annulus to the tip of the papillary muscle).

**APD of the left ventricle is ALP and PMP.**

**ALP** (annulus-papillary distances for anterolateral papillary muscle)

- G: Tip of the anterolateral papillary muscle
- A(12)G: Annulus-papillary distance measured from mid-point of the anterior leaflet of the mitral valve at 12 o’clock at the level of mitral annulus to the tip of the anterolateral papillary muscle.
- B(6)G: Annulus-papillary distance measured from mid-point of the posterior leaflet of the mitral valve at 6 o’clock at the level of mitral annulus to the tip of the anterolateral papillary muscle.
- C(10)G: Annulus-papillary distance from anterolateral commissure at 10 o’clock at the level of mitral valve annulus to the tip of the anterolateral papillary muscle.
- D(2)G: Annulus-papillary distance from posteromedial commissure at 2 o’clock at the level of mitral valve annulus to the tip of the anterolateral papillary muscle.
- E(9)G: Annulus-papillary distance from the lateral end of D_max_ at 9 o’clock at the level of mitral valve annulus to the tip of the anterolateral papillary muscle.
- F(3)G: Annulus-papillary distance from the medial end of D_max_ at 3 o’clock at the level of mitral valve annulus to the tip of anterolateral papillary muscle

**PMP** (annulus-papillary distances for posteromedial papillary muscle)

- H: Tip of the posteromedial papillary muscle
- A(12)H: Annulus-papillary distance measured from mid-point of the anterior leaflet of the mitral valve at 12 o’clock at the level of mitral annulus to the tip of the posteromedial papillary muscle.
- B(6)H: Annulus-papillary distance measured from mid-point of the posterior leaflet of the mitral valve at 6 o’clock at the level of mitral annulus to the tip of the posteromedial papillary muscle.
- C(10)H: Annulus-papillary distance from anterolateral commissure at 10 o’clock at the level of mitral valve annulus to the tip of the posteromedial papillary muscle.
- D(2)H: Annulus-papillary distance from posteromedial commissure at 2 o’clock at the level of mitral valve annulus to the tip of the posteromedial papillary muscle.
- E(9)H: Annulus-papillary distance from the lateral end of D_max_ at 9 o’clock at the level of mitral valve annulus to the tip of the posteromedial papillary muscle.
- F(3)H: Annulus-papillary distance from the medial end of D_max_ at 3 o’clock at the level of mitral valve annulus to the tip of posteromedial papillary muscle

**APD of the right ventricle:**

- Gt: Tip of the most prominent papillary muscle of the right ventricle, usually only the anterolateral papillary muscle.
- A(8)Gt: Annulus-papillary distance measured from anteroseptal commissure (AS) at 8 o’clock at the level of the tricuspid annulus to the tip of the papillary muscle.
- B(10)Gt: Annulus-papillary distance measured from the perpendicular line “a” insertion point at 10 o’clock at the level of the tricuspid annulus to the tip of the papillary muscle.
- C(12)Gt: Annulus-papillary distance measured from anterio-posterior (AP) commissure at 12 o’clock at the level of the tricuspid annulus to the tip of the papillary muscle.
- D(2)Gt: Annulus-papillary distance measured from perpendicular line “c” insertion point at 2 o’clock at the level of the tricuspid annulus to the tip of the papillary muscle.
- E(4)Gt: Annulus-papillary distance measured postero-septal commissure (PS) at 4 o’clock at the level of the tricuspid annulus to the tip of the papillary muscle.
- F(6)Gt: Annulus-papillary distance measured from perpendicular line “b” insertion point at 6 o’clock at the level of the tricuspid annulus to the tip of the papillary muscle.

**Impression material preparation**

*Preparation of* *Gypsum cement (Lunabean^TM^)*

875 ml of cold water (5.9°C) and 1kg of gypsum cement are added to a mixing bowl. Slow and gentle stirring was done with a wooden ice-cream stick to prevent the formation of air bubbles within the mixture. Once the impression material reached a smooth, white texture, casting gypsum cement into the heart was initiated. A glass funnel was placed in 1 of the openings, and gypsum cement was directly cast into the cavities until filled. The process is repeated until all chambers are filled.

*Preparation of* *EVA (Ethylene-vinyl acetate) copolymers (Stanley^TM^)*

Two glue sticks (7mm x 150mm/ 7mm x 300mm) were loaded into the glue gun for 1^st^-time use. Pointing the glue gun inferiorly via the openings created, the trigger was pulled to feed the melted glue into the heart cavities. Continuous glue injection into the heart and reloading of glue sticks are conducted until all cavities are filled to the brim. An average volume of 63.l ± 23.7 ml was used to fill the heart cavities.

*Preparation of* *RTV (Room-temperature-vulcanizing) silicone*

200 g of silicone material and 8 ml of catalyst (4% of the material) are added to a mixing bowl and stirred with A DIY stirrer (metal spoon encased in a glove). A syringe was used to facilitate the casting of RTV silicone. The impression material was poured into the syringe barrel and inserted into the heart through the openings. The plunger is pushed while lifting the syringe out of the openings to cast the impression material smoothly. More impression material is placed into the barrel, and the procedures are repeated till the heart cavities are filled. An average volume of 67.5±3.5ml was used to fill the cavities.

*Preparation of* *EasyMould^TM^ silicone rubber*

We mixed components A and B in a 1:1 ratio into a disposable plastic beaker and stirred with a DIY stirrer until a uniform light blue color. A syringe was used to facilitate the casting of RTV silicone. The impression material was poured into the syringe barrel and inserted into the heart through the openings. The plunger is pushed while lifting the syringe out of the openings to cast the impression material smoothly. More impression material is placed into the barrel, and the procedures are repeated till the heart cavities are filled. An average volume of 108.5±30.4ml was used to fill the cavities.

*Preparation of* *Dragonskin^TM^ silicone rubber*

We mixed components A and B in a 1:1 ratio into a disposable plastic beaker and stirred with a DIY stirrer till a uniform translucent white color. A syringe was used to facilitate the casting of RTV silicone. The impression material was poured into the syringe barrel and inserted into the heart through the openings. The plunger is pushed while lifting the syringe out of the openings to cast the impression material smoothly. More impression material is placed into the barrel, and the procedures are repeated till the heart cavities are filled. An average volume of 90.9±33.9ml was used to fill the cavities.

**Measurements of AV Valves**

*Measurement of the Annulus Ring*

Three measurements were taken for each section of the annulus ring, for mitral valve anterior annulus (AAC) and posterior annulus (PAC); for tricuspid valve, septal annulus (SA), anterior annulus (AA), and posterior annulus (PA), to ensure that the suture material was traced around the annulus accurately.

*Measurement of the distance from Commissures to Papillary Muscle*

Mitral valve: Two sets of measurements was taken from the respective commissure to the tip of the corresponding papillary muscles.

Tricuspid valve: Three measurements were taken for each distance from each respective commissure to the tip of the papillary muscle, from the septal commissure (SC), anterior commissure (AC), and posterior commissure (PC), to ensure that the suture was sufficiently taut for measurement.

*Measurement of the Vertical Distances for tricuspid valve*

The data obtained from the measurement of vertical distances of the septal position (VS), anterior position (VA), and posterior position (VP), were taken once when the heart was in its resting state.

*Measurement of Perpendicular Distances for tricuspid valve*

The data obtained from the measurement of perpendicular distances, from the septal position (SP), anterior position (AP), and posterior position (PP), were taken once when the heart was in its resting state.

*Measurement of the Papillary Muscle*

Tricuspid valve: Three measurements of the papillary muscle were measured, height, width, and thickness of the papillary muscle. The height ranges from 2.00cm to 3.70cm. The width ranges from 1.50cm to 2.20cm. The thickness ranges from 0.271cm to 0.729cm. The height of the papillary would be the most diverse among the hearts. The thickness of the papillary muscle would be the most consistent among the hearts.

*Other Measurements*

Other measurements taken from the coronal section were the distance from the annulus ring to the tip of the papillary muscle when the heart was rested on a flat surface. The ventricle depth was calculated by summing up the distance from the annulus ring to the tip of the papillary muscle and the height of the papillary muscle.

**3D AV Annulus Geometry**

***Mitral Valve***

The relative position of all the points of significance along the annulus A to F were noted and the percentage occurrence of each point at the position in an order from highest to lowest along each axis were identified. For the x-axis, in order from highest to lowest, C = 69%, E = 46%, A = 77%, B and D = 100%, and F = 100%. For the y-axis, D = 67%, A = 69%, C = 48%, F = 48%, E = 75%, and B = 94%. For the z-axis, A = 67%, D = 33%, C = 42%, E = 40%, and B and F = 52%. The order of points A to F along each axes are depicted in Supplementary Figures 7A,B,C. Using the percentage occurrence at each position along each axis, a 3D outline of the mitral valve was drawn out and displayed in Supplementary Figure 7D (Supplementary doc-1, table-ST-1).

***Tricuspid valve***

Similar methods were used to analyse the 3D parameters of the tricuspid valve. The percentage occurrence for the points A to F along the x-axis were, F = 50%, E = 31%, A = 71%, B and D = 86%, C = 100%. For the y-axis, A = 64%, B = 52%, F = 64%, C = 69%, E = 81%, and D = 100%. For the z-axis, F = 88%, A = 52%, E = 38%, and B, C, and D = 74%. The order of the points A to F along each axes are depicted in Supplementary Figures 7 E,F,G. Supplementary Figure 7H displays the 3D outline of the mitral valve constructed using the percentage occurrence of each point along the 3 axes (Supplementary doc-1, table-ST-2).

**Supplementary Figures**

**Figure SF-1(Supplementary Figure-1)**

Recording of the 3D object


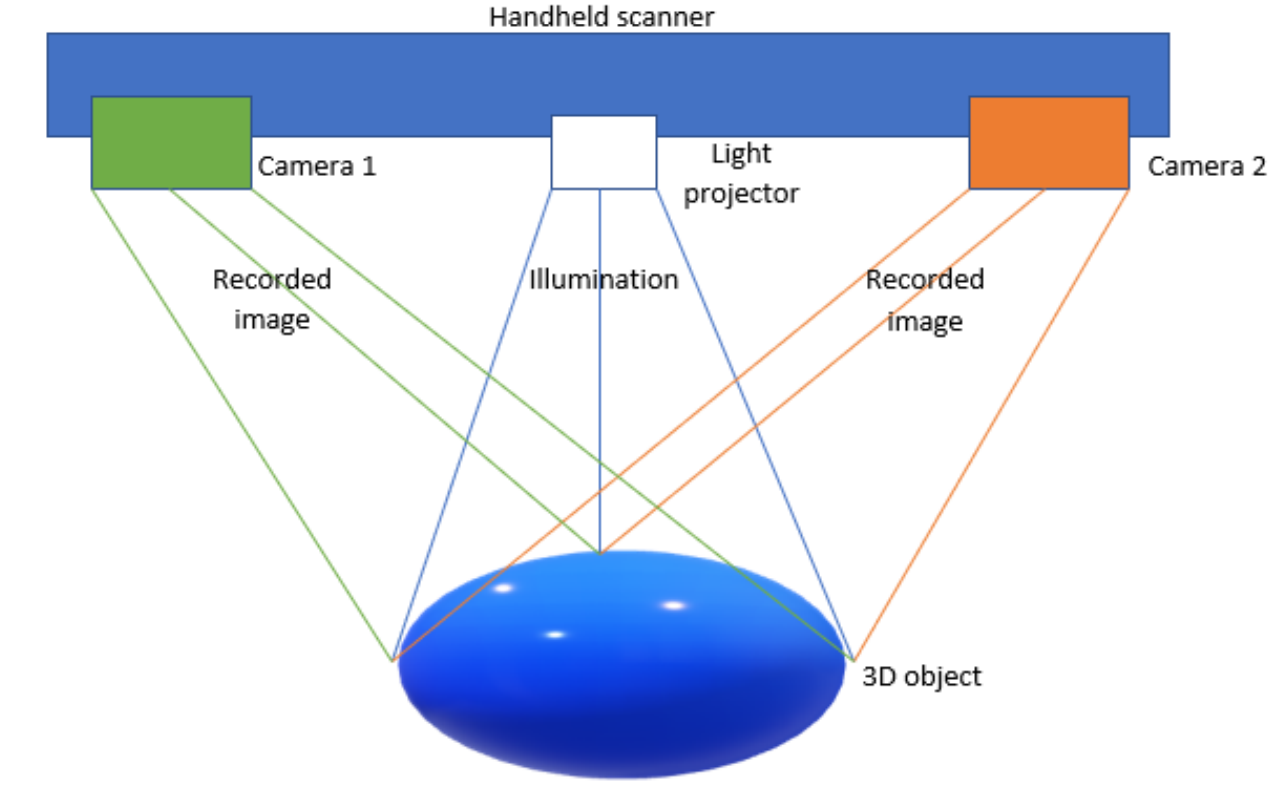

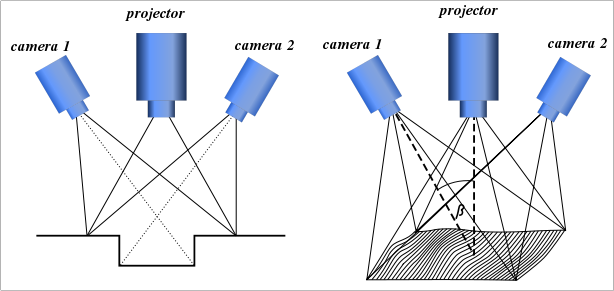


***Figure-SF1:*** *Recording of the 3D object by the handheld scanner using structured-light technology. The object is illuminated by light projected from the scanner, which is reflected into two other cameras that record the light from different angles and two different perspectives.*

**Mitral valve: Figure SF-2: (Supplementary Figure-2)**

*
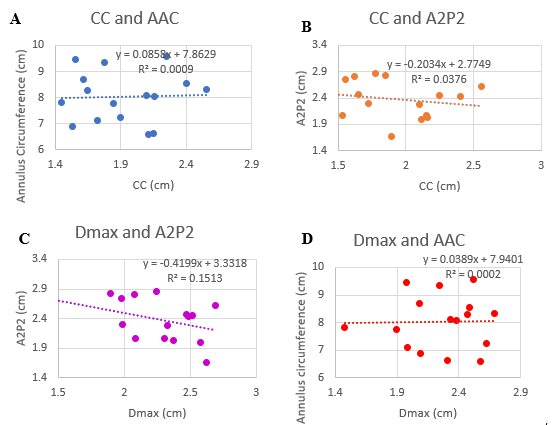
*

*Figure SF-2: Correlation between annulus parameters. (A) CC vs AAC. (B) CC vs A_2_P_2_. (C) Dmax vs A_2_P_2_. (D) Dmax vs AAC.*

**Figure SF-3: (Supplementary Figure-3)**

*
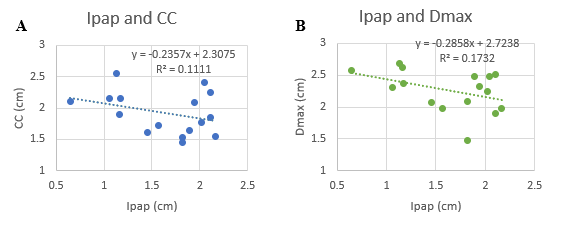
*

*Figure SF3: Correlation between Ipap and annulus parameters. (A) Ipap vs. CC. (B) Ipap vs. Dmax.*

**Figure SF-4: (Supplementary Figure-4)**

*
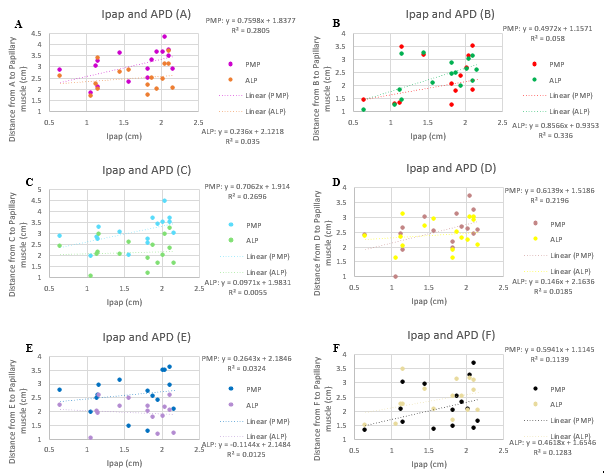
*

*Figure SF4: Correlation between Ipap and APD. (A) Ipap vs PMP and ALP for A. (B) Ipap vs PMP and ALP for B. (C) Ipap vs PMP and ALP for C. (D) Ipap vs PMP and ALP for D. (E) Ipap vs PMP and ALP for E. (F) Ipap vs PMP and ALP for F.*

**Tricuspid valve: Figure SF-5: (Supplementary Figure-5)**

*
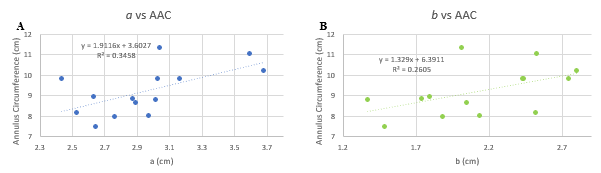
*

*Figure SF5: Correlation between tricuspid perpendicular distance and AAC. (A) a vs. AAC. (B) b vs. AAC.*

**Figure SF-6: (Supplementary Figure-6)**

*
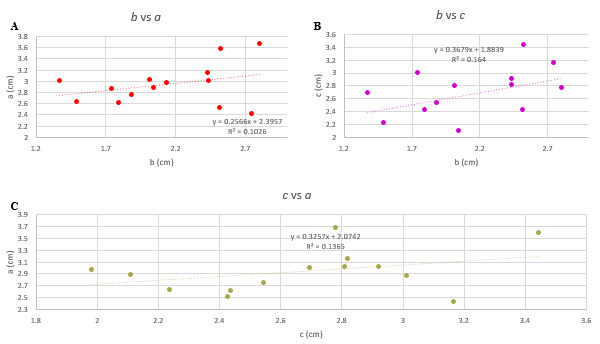
*

*Figure SF6: Correlation between tricuspid perpendicular distances. (A) b vs a. (B) b vs c. (C) c vs a.*

**Figure SF-7: (Supplementary Figure-7)**


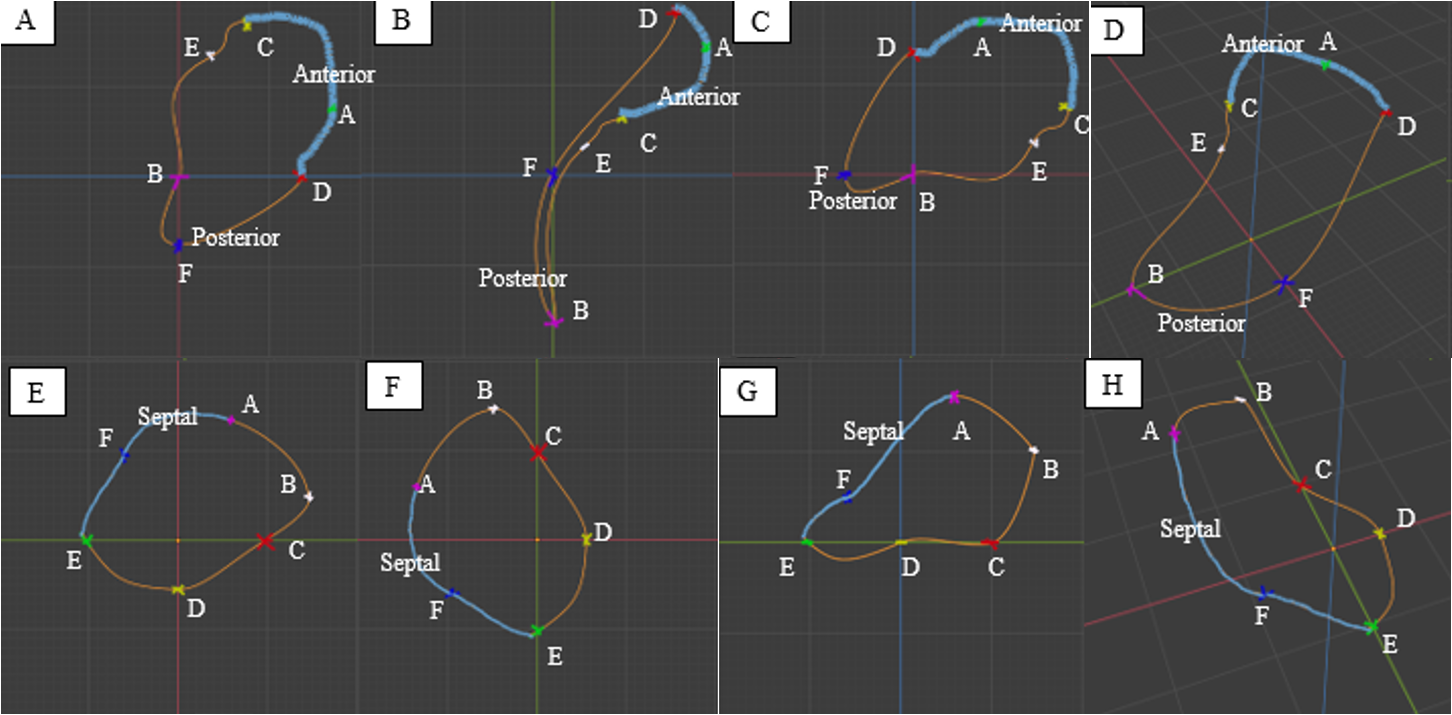


*Figure SF7: Mitral valve (A-D), Tricuspid valve (E-H), the x-axis, y-axis, and z-axis are represented by the colors red, green, and blue, respectively. Order of points A-F in the mitral valve along the (A) x-axis, (B) y-axis, and (C) z-axis. (D) 3D mitral valve outline from percentage occurrences of points A-F. Order of points A-F in the tricuspid valve along the (E) x-axis, (F) y-axis, and (G) z-axis. (H) 3D tricuspid valve outline from the percentage occurrences of points A-F.*
